# The Close of Volume XVI

**Published:** 1895-12

**Authors:** 


### THE CLOSE OF VOLUME XVI.

THE close of the year's work on a journal such as this necessarily leads to reflection. The labor of preparing a volume of eight hundred pages is not a trifling matter, and is in itself worthy of notice, but the important consideration for the editor is that, in overlooking the work of the past, he can dwell with peculiar satisfaction upon the energy in societies and activity in individuals manifested in its well-filled pages. It is with some degree of pride, therefore, that we can consistently feel that this journal has in the past year maintained its well-earned character, and for this we specially desire to thank our contributors, who have so unswervingly aided in the support of a journal devoted to the highest interests of the profession of dentistry.

The support given it by the large list of subscribers, now extended and constantly increasing over all civilized lands, is a silent but most effective testimony to the value of the journal as a medium for professional thought, and we naturally feel gratified at the result.

The year, now nearly closed, has been one that followed several of great business depression, but the past year has largely and happily changed this, and the Journal, in common with other enterprises, has been benefited thereby.

It is hoped that the dental profession will bear in mind that the only way to raise to a higher standard is to work persistently for it, and this can always best be done through that medium that will educate the largest number. For this purpose no agency is so effective as the well-conducted journal. It is desired that those who have been the gainers by the labor devoted to the production of this periodical will endeavor to increase its influence by individual effort.

The lapse of a year is too short a time to note marked changes, yet it must seem to the reflecting mind that there have been influences at work during the past twelve months that must result in a healthful growth in the future. Among these can be noted the tendency towards a more scientific trend of thought, with a corresponding lessening of that crude and superficial self-assertion so prevalent. For these beginnings of a more fruitful era the profession of dentistry is to be congratulated. These are the guide-posts that unerringly point to a future where essays will be founded on exact knowledge and not on mere speculation.

With the promises of the past we enter with confidence into another year, feeling that the work completed is bearing rich fruit, and that this will continue to be the result as long as men are true to their highest aspirations.

